# Supplementary figures and images for: Effects of Lactobacillus salivarius LN12 in Combination with Amoxicillin and Clarithromycin on Helicobacter pylori Biofilm In Vitro
Source: Microorganisms. 2021 Jul 28;9(8):1611. doi: 10.3390/microorganisms9081611 (PMC8399496; doi:10.3390/microorganisms9081611)

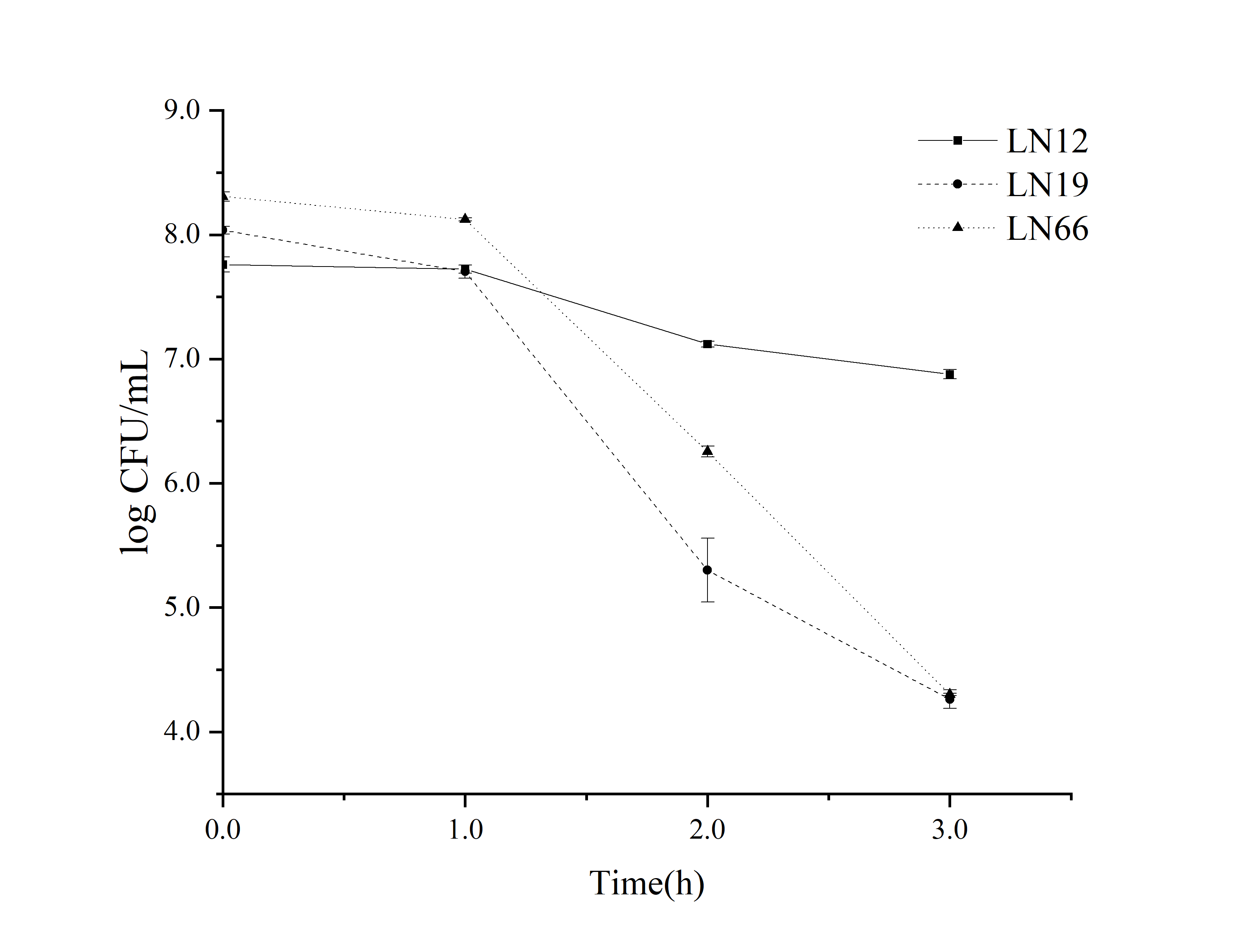

Supplement: Supplementary file 1 [file microorganisms-09-01611-s001.zip › Supplementary Figure S1.tif]

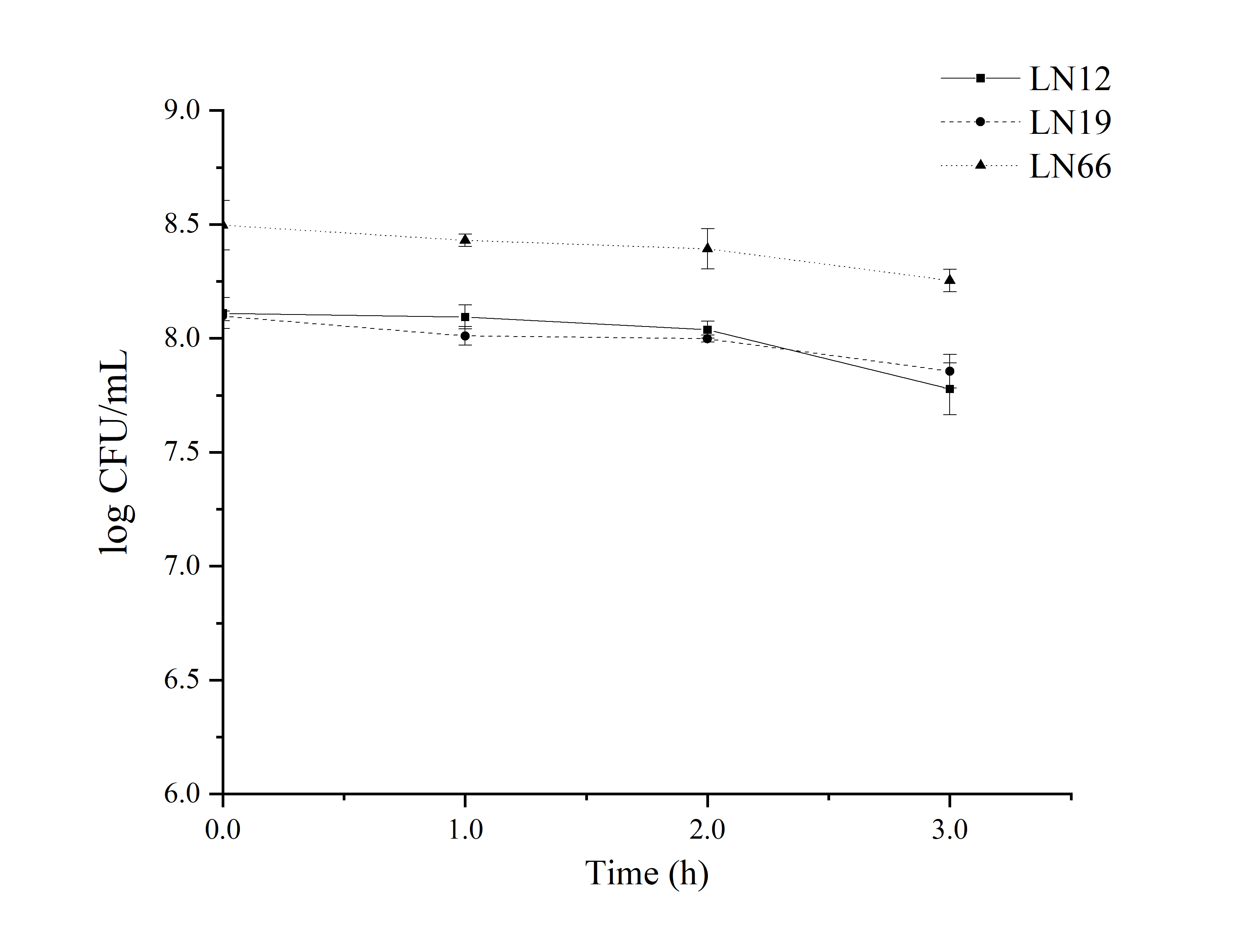

Supplement: Supplementary file 1 [file microorganisms-09-01611-s001.zip › Supplementary Figure S2.tif]

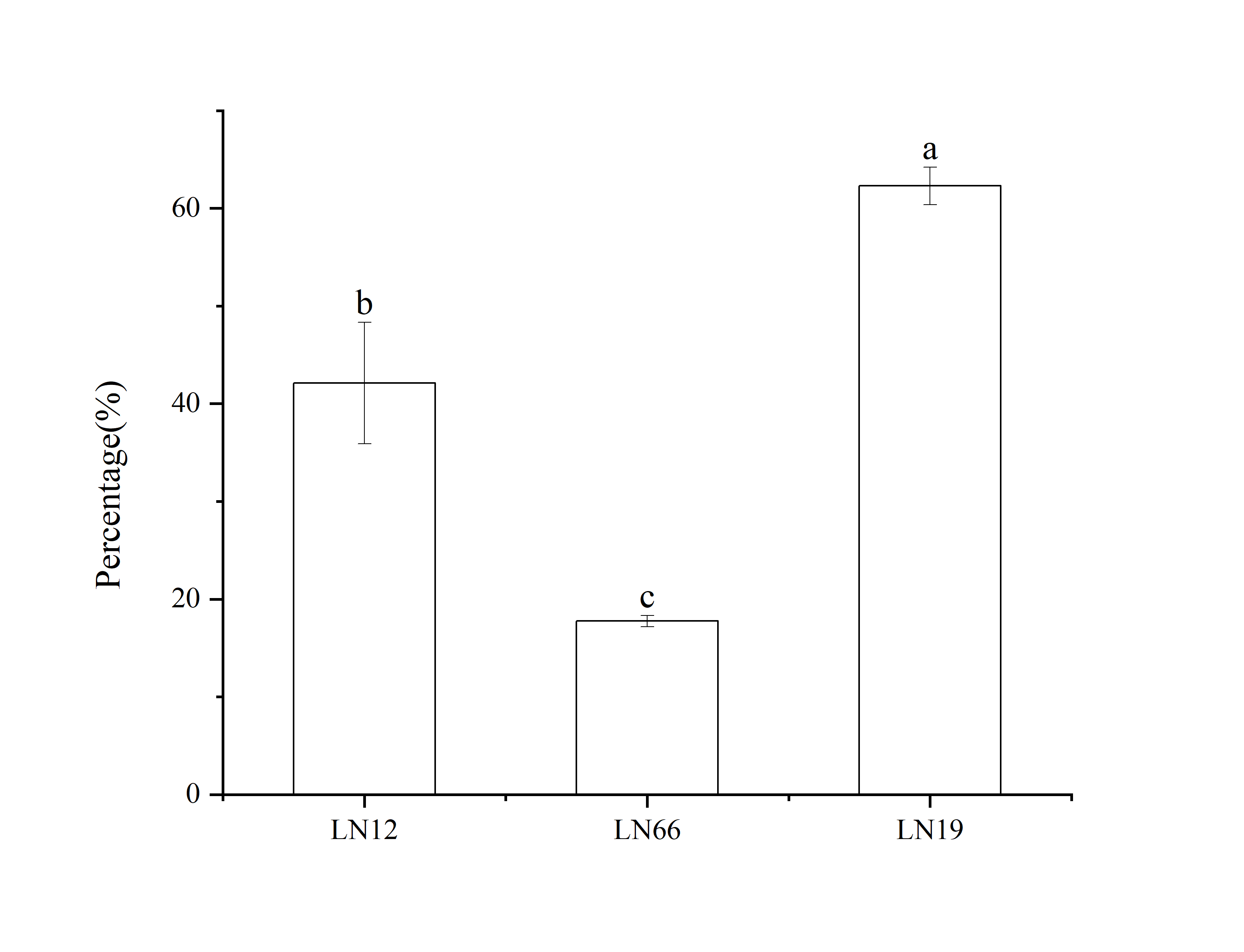

Supplement: Supplementary file 1 [file microorganisms-09-01611-s001.zip › Supplementary Figure S3.tif]

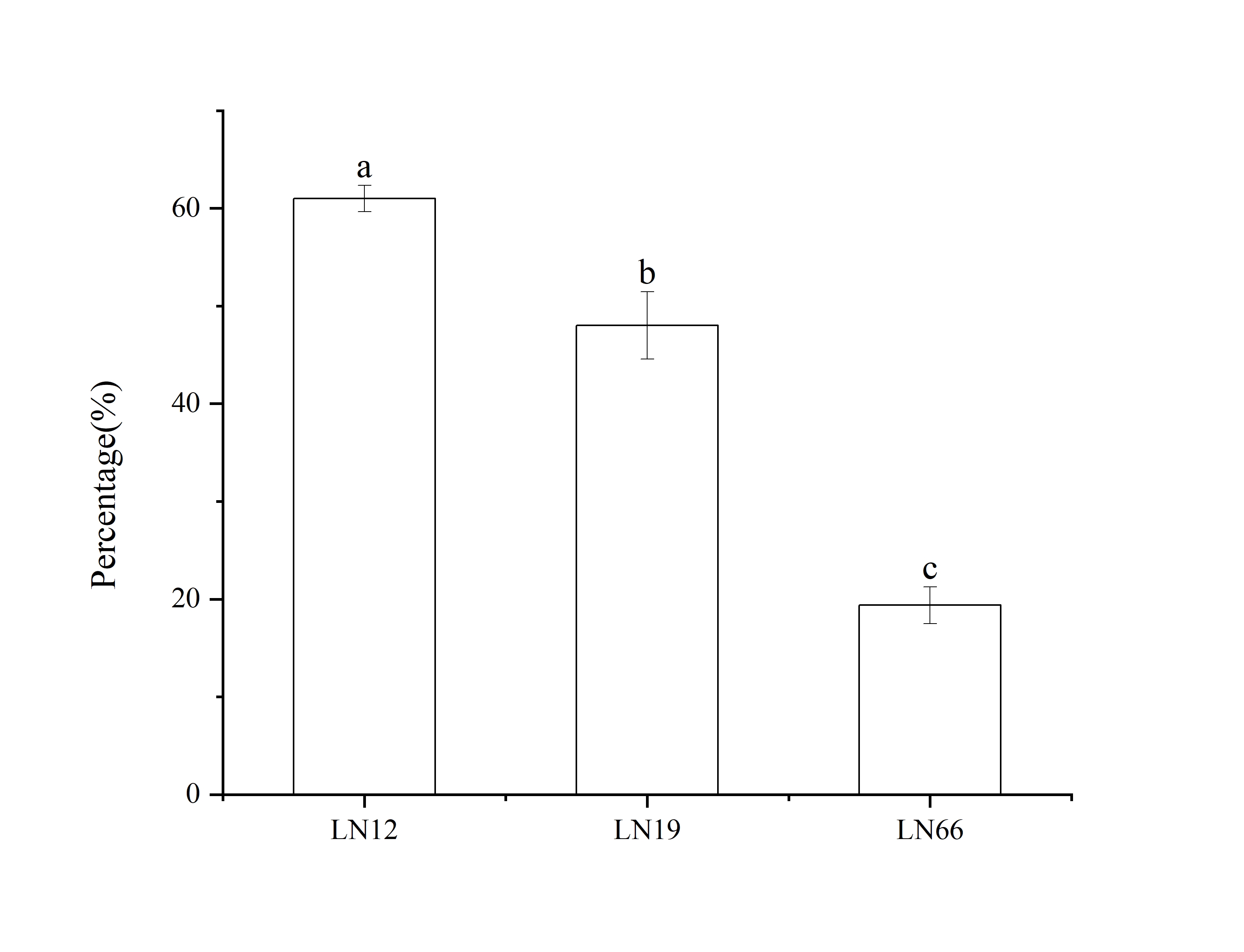

Supplement: Supplementary file 1 [file microorganisms-09-01611-s001.zip › Supplementary Figure S4.tif]
